# Medical History from the Earliest Times

**Published:** 1892-07-16

**Authors:** E. T. Withington


### Medical History from the Earliest Times.

By E. T. Withington, M.A., M.B. Oxon.

### XVII.—EARLY ROMAN MEDICINE.

"Rome," says Pliny, "was for six centuries without physicians, but not without physic," and in his "Natural History" he gives us a copious account of the mixture of simples and superstition of which this physic was composed. But Pliny is a prejudiced witness; his ideal is the ancient Roman of the good old days, whose small farm supplied him with food and with all the medicines which his hardy frame required, and his object is to show that empire, luxury, and physicians all came in about the same time. Yet even by his own account, the first Greek practitioner came to Rome in the year of the city 535, and other writers mention "medici" at a much earlier date.

The native Roman medicine may be briefly dismissed. King Numa is said to have declared that all diseases come from the gods, and are to be averted by prayer and sacrifice. The number of medical divinities was very great, for besides Apollo and Minerva, temples were dedicated to Febris, Mephitis, and even, it is said, a Dea Scabies, while the young mother might appeal to no less than fourteen goddesses, from Juno Lucina down to Prosa and Portvorta.

In later times this primitive medicine was modified in two directions by the magic of Etruria and by the more rational practice of the Greek colonies in South Italy, and indications of both these influences are seen in the works of Pliny's favourite hero, Cato the Censor, who hated all Greeks, and especially hated Greek physicians. In his book on "Agriculture," Cato gives directions for treating sick slaves and cattle, of which the latter are most important. For oxen he prescribes a remedy in which three is the ruling number—three grains of salt, three laurel leaves, three leaves of rue, &c., to be given three times a day for three days; both the animal and the giver of the drug must be fasting, and both must stand erect. Of like nature are the incantations which he recommends for cases of fracture and dislocation.

But Cato's grand panacea is cabbage, the favourite vegetable of the Pythagoreans. He gives it internally, both raw and cooked; he applies it as a poultice to sores, declaring that it will even cure cancer; and he squirts its juice into sinuses and fistulæ by means of a syringe composed of a bladder tied to a reed. If a slave is ill and cabbage will not cure him he should be got rid of, for it is bad economy to feed men who cannot work. That this hint was not neglected is evident from a decree of the Emperor Claudius, which provides that sick slaves exposed by their masters shall be free if they recover, and that the killing of a sick slave shall be murder. There is, however, one department of the healing art in which the Romans have some claim to originality, that of state medicine and hygiene. Thus the "lex regia" of King Numa ordered the performance of Cæsarian section on women who died in child-bed, while the law of the "Twelve Tables" committed the madman (furiosus) to the care of his nearest relatives, fixed the extreme duration of pregnancy at 300 days, and forbade the burning or burial of bodies within the City walls. One of the most ancient remnants of Roman masonry is the great sewer, or "Cloaca maxima," ascribed by Livy to the Tarquins. Recent excavations have revealed special spots set apart for burning refuse, and a pillar has been found with the inscription, "Take your rubbish further, or you'll be fined." Above all, the 14 great aqueducts, which were at one time capable of supplying the city with more than three hundred million gallons of drinkable water daily, show that in some forms of sanitation, ancient Rome need not have feared comparison with modern London. With a few doubtful exceptions such as these, it must be remembered that the medicine now to be described is entirely Greek, and, indeed, that everything worthy of the name, from Hippocrates to Harvey, is Greek medicine, whether we find it at Alexandria, Rome, Bagdad, Salerno, or Paris.

According to Pliny, the first Greek practitioner came to Rome B.C. 219, and had the appropriate name of Archagathus (a good beginning). He was received with favour, granted the citizenship and a surgery in the Acilian crossway, and entitled "Vulnerarius," the Wound-curer. But the people were soon disgusted "by his ferocity in cutting and burning," so they called him the Executioner (carnifex), and would have no more of him. No Greek writer knows anything of Archagathus, and he was not improbably a low-class surgeon, who, having failed at home, thought he might still make his fortune among the "barbarians."

The next arrival was of very different character, Asclepiades, of Prusa, probably the most successful practitioner of antiquity. Beginning as a poor man, he may have learned medicine at Alexandria, and certainly studied rhetoric at Athens, where he was possibly that Asclepiades of whom Athenæus says that he followed the philosophers in the mornings and earned his bread as a miller's labourer in the afternoons. Finally, he came to Rome, where his high culture and wonderful address gained him the friendship of Crassus and Cicero, and made him the fashionable physician of the day. But Asclepiades had other aids to success in addition to "a good bedside manner." He introduced a new theory into medicine based upon the atomic philosophy of Democritus and Epicurus, a doctrine about this time made popular in Rome by the great poem of Lucretius. He declared that the human body was composed of atoms, with intervals between them forming canals or "pores," through which still finer atoms circulated, and rejecting the humoral doctrines, he ascribed disease to changes in the relation of the atoms and pores, and especially to a blocking up of the latter.

Secondly, Asclepiades abused his predecessors, a never-failing recipe for notoriety in medicine. He called the expectant treatment of Hippocrates "a meditation on death," ridiculed the "vis medicatrix," asserting that Nature did quite as much harm as good, and that the physician should not be her servant but her master, and should at once intervene in order to cure his patient "quickly, safely, and pleasantly." He was the first to distinguish acute and chronic diseases, and paid great attention to the latter, which were naturally prominent in a large and luxurious community, and which had been somewhat neglected by the Hippocratic school.

Thirdly, Asclepiades employed modes of treatment well adapted to his patients. Rejecting emetics and violent purgatives, he relied chiefly on diet, rubbing, wine, and passive exercise, prescriptions suited to his more Epicurean clients, and upon active exercise, and cold water, including the recently-invented shower-bath, which would attract those anxious to gain the ancient Roman hardihood. With such qualifications it was hardly necessary that Asclepiades should also have restored an apparently dead person to life to achieve a brilliant professional success.

His actual merit is more difficult to estimate, for his writings have perished, and his critics, as might be expected, give opposing verdicts, but on the whole the judgment is favourable. Celsus confesses himself much indebted to Asclepiades; Pliny, though indignant that "a poor man, sprung from the most volatile of nations, should give laws of health to mankind," cannot withhold his admiration. Apuleius calls him a second Hippocrates, and if Galen judges more severely, it is partly because he cannot forgive him for attacking the Father of Medicine, and for being in some sense the precursor of the hated Methodists. A modern writer has called Asclepiades "the Hippocrates of chronic disease." This is, perhaps, excessive, but if not to be compared to the Father of Physic, he may be safely called the Father of Fashionable Physicians.

